# *Hepatozoon silvestris* sp. nov.: morphological and molecular characterization of a new species of *Hepatozoon* (Adeleorina: Hepatozoidae) from the European wild cat (*Felis silvestris silvestris*)

**DOI:** 10.1017/S0031182016002316

**Published:** 2016-12-12

**Authors:** ADNAN HODŽIĆ, AMER ALIĆ, SENAD PRAŠOVIĆ, DOMENICO OTRANTO, GAD BANETH, GEORG GERHARD DUSCHER

**Affiliations:** 1 Department of Pathobiology, Institute of Parasitology, University of Veterinary Medicine Vienna, Veterinaerplatz 1, 1210 Vienna, Austria; 2 Department of Pathology, Faculty of Veterinary Medicine, University of Sarajevo, Zmaja od Bosne 90, 71000 Sarajevo,Bosnia and Herzegovina; 3 Department of Veterinary Medicine, University of Bari, Str. prov. per Casamassima km 3, 70010 Valenzano, Italy; 4 Koret School of Veterinary Medicine, Hebrew University of Jerusalem, P.O. Box 12, 76100 Rehovot, Israel

**Keywords:** *Hepatozoon silvestris* sp. nov., *Hepatozoon felis*, European wild cat, *Felis silvestris silvestris*, morphology, molecular characterization, Bosnia and Herzegovina

## Abstract

Based on morphological and genetic characteristics, we describe a new species of *Hepatozoon* in the European wild cat (*Felis silvestris silvestris*), herein named *Hepatozoon silvestris* sp. nov. The study also provides the first data on the occurrence of *H. felis* in this wild felid. *Hepatozoon* meronts were observed in multiple cross-sections of different organs of four (44%) cats. Additionally, extracellular forms, resembling mature gamonts of *Hepatozoon*, were found in the spleen and myocardium of two cats. Furthermore, tissues of six animals (67%) were positive by PCR. *Hepatozoon felis* was identified infecting one cat (11%), whereas the 18S rRNA sequences of the remaining five cats (56%) were identical, but distinct from the sequences of *H. felis*. Phylogenetic analyses revealed that those sequences form a highly supported clade distant from other *Hepatozoon* spp. Future studies should include domestic cats from the areas where the wild cats positive for *H. silvestris* sp. nov. were found, in order to investigate their potential role to serve as intermediate hosts of this newly described species. Identification of its definitive host(s) and experimental transmission studies are required for elucidating the full life cycle of this parasite and the possible alternative routes of its transmission.

## INTRODUCTION

Species of the genus *Hepatozoon* Miller (1908) are apicomplexan parasites that infect a wide range of vertebrate intermediate hosts (i.e. mammals, birds, reptiles, amphibians, marsupials), whereas haematophageous invertebrates (e.g. ticks, mosquitoes, mites, sand flies, fleas) may serve both as vectors and definitive hosts of these organisms (Smith, 1996). All species of *Hepatozoon* share a life cycle that involves sporogonic development and oocyst formation in the invertebrate host, and merogony and gametogony that occur in vertebrate hosts. Ingestion of the invertebrate host containing mature oocysts by the vertebrate host represents a main route of infection, although transplacental transmission and transmission by predation have also been reported (Murata *et al.* 1993; Johnson *et al.* 2009; Baneth *et al.* 2013). In addition to the meront stages, the occurrence of small tissue cysts containing single or multiple parasitic stages (cystozoites) have been described in several species of *Hepatozoon* emphasizing the complexity of their life cycles (Smith, 1996). Tissue cysts are considered to be associated with parasite transmission by predation (Smith *et al.* 1994; Vincent-Johnson *et al.* 1997; Paperna *et al.* 2002; Baneth and Shkap, 2003; Baneth *et al.* 2007).

To date more than 340 species of *Hepatozoon* have been described with less than 50 of them in mammals, but a complete and documented life cycle is available only for few of them (Smith, 1996; Baneth, 1998). Several studies have also suggested that *Hepatozoon* may not be a single genus as it contains some distinct lineages (Kvičerová *et al*. 2014; Karadjian *et al.* 2015); however, division of the genus is considered controversial (Maia *et al.* 2016). The parasite in felids was first described in India and initially named *Leucocytozoon felis domestici* (Patton, 1908). Thereafter, it was transferred to the genus *Hepatozoon* due to the high morphological similarity of gamont stages found in the blood of cats to those of *Hepatozoon canis*, which mainly infects domestic and wild canids (Wenyon, 1926). Since then, *Hepatozoon* spp. have been reported in domestic cats and various species of wild cats, and it is assumed that two or more species could be found in felids worldwide (Criado-Fornelio *et al.* 2006; Kubo *et al.* 2010; Pawar *et al.* 2012; Baneth *et al.* 2013; Tateno *et al.* 2013). However, the parasite in cats was also referred to as *Hepatozoon*-like or *Hepatozoon* spp., without committing to a certain species, as the studies which described it were based only on morphological or genetic descriptions (Klopfer *et al.* 1973; Baneth *et al.* 1998; Perez *et al.* 2004; Rubini *et al.* 2006; Metzger *et al.* 2008; Ortuño *et al.* 2008; Salakij *et al.* 2008). Thereafter, the organism was finally morphologically and genetically characterized as *Hepatozoon felis* and recognized as the predominant agent of feline hepatozoonosis (Baneth *et al.* 2013).

The European wild cat (*Felis silvestris silvestris*) is the most common wild felid species in Europe, with populations living throughout the continent, and thus classified in the category of ‘Least Concern’ in the International Union for Conservation of Nature (IUCN) Red List of Threatened Species (Yamaguchi *et al.* 2015). In Bosnia and Herzegovina, wild cats inhabit the northeastern part of the country, and areas along the northern and eastern border where they live in sympatry with domestic cats (Yamaguchi *et al.* 2015). In the past few years, the interest of the scientific community in wild cats has increased as they have been recognized as natural reservoirs and potential source of pathogens for domestic cats (Falsone *et al.* 2014; Gallusová *et al.* 2016; Veronesi *et al.* 2016*a*, *b*).

In the present study, we describe a new species of *Hepatozoon* in the European wild cat, based on morphological and genetic characteristics, herein designated as *Hepatozoon silvestris* sp. nov. Moreover, the study also provides the first data on the occurrence of *H. felis* in this wild felid.

## MATERIALS AND METHODS

### Sample collection

From 2011 to 2016, carcasses of nine adult European wild cats were collected in five municipalities of northwestern (Bihać, Bosanski Petrovac), northern (Odžak), eastern (Goražde) and central (Gornji Vakuf) Bosnia and Herzegovina (Table 1). All animals were killed by hunters and delivered for necropsy to the Department of Pathology of the Faculty of Veterinary Medicine in Sarajevo. At necropsy, carcasses were inspected for the presence of ticks, and origin, sex and age of each animal were recorded. Duplicate tissue samples (heart, lung, spleen, bone marrow, lingual muscle, diaphragm muscle, brachial muscle, masseter muscle) were collected, and either stored at −20 °C until processed or fixed in 10% neutral-buffered formalin for up to 24 h. However, not all the tissues were sampled from each wild cat. Ticks collected from the animals were stored in 70% ethanol and were sent along with frozen tissue samples to the Institute of Parasitology, University of Veterinary Medicine, Vienna, Austria for species identification (Estrada-Peña *et al.* 2004) and molecular analysis, respectively.

**Table 1.** Origin, sex and age of the European wild cats (*Felis silvestris silvestris*) collected in this study. Only tissues tested by both PCR and histopathology are included in the table.

ID No.	Location in Bosnia and Herzegovina	Sex	Age	Heart		Lung		Spleen		Skeletal muscle		Species
				Histology	PCR	Histology	PCR	Histology	PCR	Histology	PCR	
53/11	Goražde	F	adult	–	neg	–	neg	–	N/A	–	N/A	Negative
382/15	Bosanski Petrovac	M	adult	–	neg	–	neg	–	neg	–	neg	Negative
431/15	Gornji Vakuf	F	adult	–	pos	–	N/A	–	N/A	–	N/A	*Hepatozoon silvestris* sp.nov.
115A/16	Bosanski Petrovac	F	adult	–	pos	–	neg	–	pos	–	neg	*H. silvestris* sp.nov.
115B/16	Bosanski Petrovac	M	adult	–	neg	–	neg	–	neg	–	N/A	Negative
115C/16	Bosanski Petrovac	M	adult	–	neg	–	pos	–	neg	–	neg	*H. silvestris* sp.nov.
115D/16	Bosanski Petrovac	F	adult	+	pos	–	pos	–	N/A	+	pos	*H. silvestris* sp.nov.
152/16	Bihać	M	adult	+^a^	pos	–	pos	–^a^	pos	–	neg	*H. silvestris* sp.nov.
170/16	Odžak	F	adult	+++	pos	+++	pos	+^a^	pos	+	pos	*Hepatozoon felis*

N/A, not applicable, because tissue was not collected; PCR: pos, positive; neg, negative.

Histopathology: (−) negative, (+) mild, (++) moderate, (+++) severe degree of infection according to Kubo *et al.* (2006).

a tissue samples positive by cytology.

### Pathological and microscopic examination

For histopathology, formalin-fixed tissue samples were routinely processed, embedded in paraffin and cut at 3–6 µm sections. Sections were deparaffinized and stained with haematoxylin and eosin (H&E). In addition, multiple spleen and heart touch imprints from each animal were made on glass slides, stained with May–Grunwald–Giemsa and examined for the presence of parasitic forms. The degree of *Hepatozoon* parasitic load in tissue sections was graded according to Kubo *et al*. (2006) as negative (−), mild (+), moderate (++) and severe (+++). The presence of *Hepatozoon* oocysts in ticks collected was examined in haemolymph smears as previously described (Baneth *et al.* 2007). Developmental stages found in this study were measured with a calibrated ocular micrometre and sizes were expressed in micrometres (*μ*m) as mean ± s.d. (× ± s.d.).

### DNA extraction, PCR amplification and sequencing

Total DNA was extracted from 15 to 20 mg of frozen heart, lung, spleen and brachial muscle tissue using the High Pure PCR Template Preparation Kit (Roche Diagnostics, Germany) and following the manufacturer's instructions. Paraffin-embedded tissues of three cats positive by histopathology (115A/16, 152/16, 170/16) were deparaffinized and subjected to DNA extraction using the same kit. However, from cat no. 170/16 there were only formalin-fixed samples.

A 620 bp fragment of the 18S rRNA gene of *Hepatozoon* spp. was amplified using the genus-specific primer set H14Hepa18SFw 5′-GAAATAACAATACAAGGCAGTTAAAATGCT-3′ and H14Hepa18SRv 5′-GTGCTGAAGGAGTCGTTTATAAAGA-3′ (Hodžić *et al.* 2015). Amplification was conducted under the following conditions: 95 °C for 2 min followed by 35 cycles of 95 °C for 1 min, 58 °C for 1 min, 72 °C for 1 min. Final extension was performed at 72 °C for 5 min. The PCR was carried out in a final volume of 25 *µ*L using 5 × Green Reaction Buffer and GoTaq G2^®^ Polymerase (Promega, Germany). The DNA from tissue samples positive by PCR were additionally tested using primers HAM-1F 5′-GCCAGTAGTCATATGCTTGTC-3′ and HPF-2R 5′-GACTTCTCCTTCGTCTAAG-3′, which amplify approximately 1700 bp long fragment of 18S rRNA gene of *Hepatozoon* spp. (Criado-Fornelio *et al.* 2006), and obtained sequences were used for phylogenetic analyses. This PCR was processed under the following conditions: 95 °C for 2 min, then 35 cycles of 95 °C for 30 s, 58 °C for 30 s, 72 °C for 1·5 min and finally 72 °C for 5 min. The second PCR assay was performed in a final volume of 25 *µ*L using Ilustra™ Hot Start Mix Ready-To-Go (GE Healthcare Europe GmbH, Germany). DNA from a fox positive for *H. canis* was used as positive control in both PCRs.

To rule out possible misdiagnosis of *Hepatozoon* tissue forms with *Toxoplasma gondii* and *Sarcocystis* spp., DNA extracts from all tissue samples were tested for these pathogens using primers and PCR protocols published elsewhere (Homan *et al.* 2000; Kolenda *et al.* 2015).

PCR products were visualized by electrophoresis on 2% agarose gel stained with Midori Green Advance DNA stain (Nippon Genetics Europe, Germany) and sequenced in both directions by a commercial company (LGC Genomics, Germany). Sequences obtained were edited with the BioEdit software v.7.2.5 (Hall, 1999) and compared for similarity with those available in GenBank^®^ using Basic Local Alignment Search Tool (BLAST) analysis (http://www.ncbi.nlm.nih.gov/BLAST).

### Phylogenetic analyses

For the construction of a phylogenetic tree, long 18S rRNA nucleotide sequences obtained in the present study (1669 bp) were compared with the *Hepatozoon* spp. sequences deposited in the GenBank^®^ database. Only the sequences with at least 945 bp overlapping were used for the overall alignment. The sequences were aligned by the MUSCLE algorithm (Edgar, 2004) and trimmed with trimAl v.1.2 tool (Capella-Gutiérrez *et al.* 2009). Sequence analyses were performed using the Maximum Likelihood (ML), Neighbor-Joining (NJ) and Maximum Pasrimony (MP) algorithms implemented with the bioinformatics software program MEGA v.7.0 (Kumar *et al*. 2016). A model test was used to determine the most suitable nucleotide substitution model and the T92 + G (Tamura 3-parameter) was the best fitting model according to AICc values (Akaike information criterion corrected). Phylogenetic trees were constructed with MEGA v.7.0 (Kumar *et al*. 2016) and tree topology (ML, MP) were completed using the Subtree Pruning and Regrafting heruistic model. Internal nodes of the tree were estimated with 1000 bootstrap replicates. Phylogenetic network was calculated with the short 18S rRNA sequences herein generated (572 bp) and *H. felis* sequences from domestic and wild cats available in GenBank^®^. The sequences of both datasets were aligned using MUSCLE (Edgar, 2004) and the phylogenetic network was calculated with Network software v.4.6.0.0 (fluxus-engineering.com) applying the default settings. Unnecessary median vectors were reduced using MP option.

### Ethical statement

All animals used in the present study were killed by licensed hunters in accordance with the Game law of Bosnia and Herzegovina (‘OJ BiH’, no. 4/06).

## RESULTS

### Histopathological and microscopic findings

**Fig. 1.**
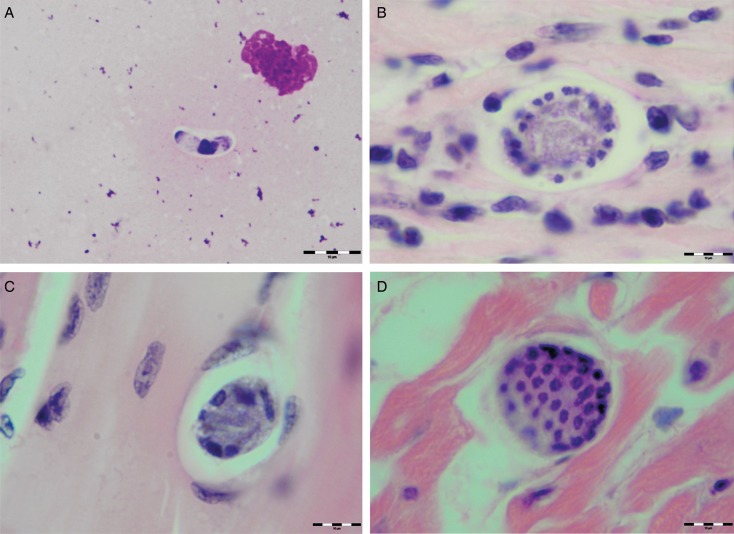
Developmental stages of *Hepatozoon silvestris* sp. nov. in the myocardium and skeletal muscle of the European wild cat. (A) Extracellular gamont-like stage from the heart touch imprint. May–Grunwald–Giemsa stain. (B) Typical wheel spoke shaped meront with micromerozoites arranged in a circle around the basal material mass. Note the mild cellular infiltrate around the meront in the heart section. (C) Developing meront with elongated circularly aligned macromerozoites in the skeletal muscle. (D) Mature meront filled with numerous merozoites. H&E stain (B–D).

**Fig. 2.**
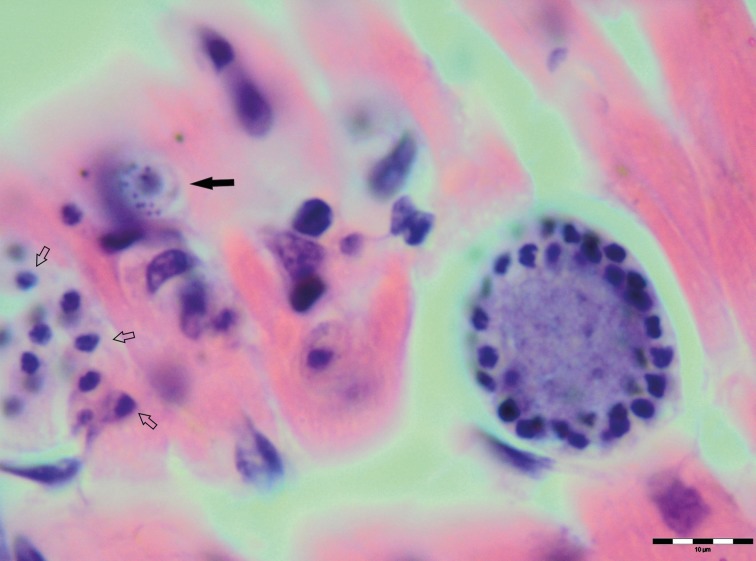
Meronts of *Hepatozoon silvestris* sp. nov. in a myocarium section. An early meront with small nuclei surrounding a residual body (arrow), wheel spoke shaped meront with circularly arranged micromerozoites, and merozoites released from ruptured meront (open arrows). H&E stain.

**Fig. 3.**
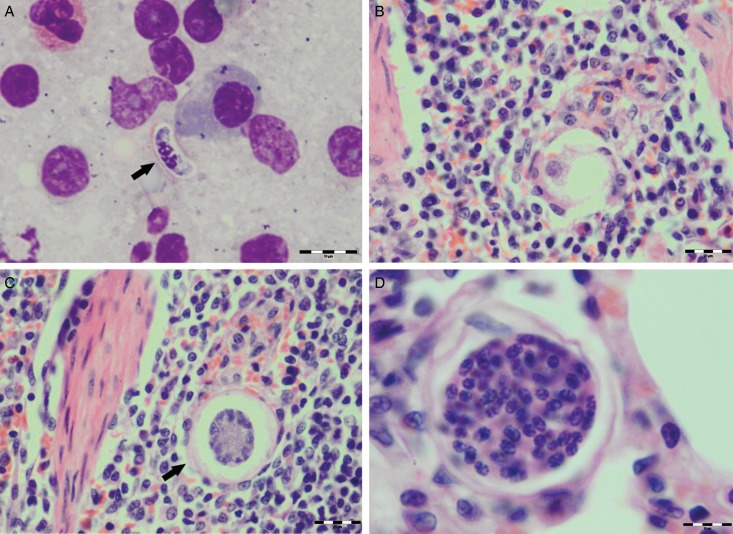
Developmental stages of *Hepatozoon felis* in the spleen and lung of the European wild cat. (A) Extracellular gamont-like stage (arrow) from a spleen touch imprint. May–Grunwald–Giemsa stain. (B) An early meront in the spleen. (C) Wheel spoke shaped meront with triangular micromerozoites in the spleen. Note the width of external capsule (arrow). (D) Mature meront in the lung section. H&E stain (B–D).

Various developmental stages of *Hepatozoon* meronts were observed in multiple cross-sections in the myocardium, lungs, spleen and skeletal muscle tissue of four (44%) out of nine European wild cats (Table 1, Figs 1, 2 and 3). These included early meronts with or without small nuclei surrounding a residual body (Figs. 2 and 3B), developing meronts containing micromerozoites circularly aligned along the meront wall (Figs 1B, 2 and 3C), meronts with larger peripherally arranged or dispersed macromerozoites (Fig. 1C), mature meronts with numerous merozoites irregularly scattered (Figs 1D and 3D), and ruptured meronts releasing merozoites (Fig. 2). Developing and mature meronts were the most common forms encountered, and the myocardium was the most frequently affected tissue among the tissues selected (Table 2). None of the structural *Hepatozoon* stages was found in the tongue, masseter, diaphragm or bone marrow. Three of the animals positive by histopathology were mildly parasitized (up to five meronts per slide), whereas numerous meronts were observed in the lungs (>50 meronts per slide) and myocardium (>35 meronts per slide) of one cat (Table 2). Extracellular gamont-like forms were observed in May–Grunwald–Giemsa stained touch imprints of the spleen and myocardium of two cats (Figs 1A and 3A, Table 2). Substantial differences in appearance, shape and size of the forms found in the cats were apparent, suggesting the existence of two different parasite species. Briefly, size of the meront and gamont-like stages, width of the capsule surrounding the meronts, as well as arrangement, size and shape of merozoites within the wheel spoke-shaped meronts were the most evident differences (Table 2, Figs 1–3).

**Table 2.** Measurements (*μ*m) of developmental stages of *Hepatozoon silvestris* sp. nov and *Hepatozoon felis* or closely relates species found in domestic and wild cats.

Hepatozoon spp.	Intermediate host	Country	Gamont	Gamont nucleus	Meront	Meront capsule (width)	Merozoite	Merozoite nucleus	Reference
*H. silvestris* sp. *nov.*	*Felis silvestris silvestris*	Bosnia and Herzegovina	11·7 ± 0·5 × 5·2 ± 0·7^a^	6·3 ± 1·3 × 3·0 ± 0·8^a^	31·7 ± 4·2 × 22·0 ± 4·6	1·1 ± 0·3	Micromerozoite: 4·2 ± 1·1 × 2·5 ± 0·4; Macromerozoite: 6·0 ± 1·0 × 3·2 ± 0·7	Micromerozoite: 2·6 ± 0·3 Macromerozoite: 4·0 ± 0·3	Present study
*H. felis*	*F. s. silvestris*	Bosnia and Herzegovina	10·5 ± 0·4 × 4·4 ± 0·4^a^	4·7 ± 0·3 × 4·4 ± 0·3^a^	36·4 ± 5·1 × 31·3 ± 5·1	1·4 ± 0·3	7·1 ± 1·0 × 2·5 ± 0·5	2·6 ± 0·2	Present study
*H. felis*	*Felis catus*	Israel	10·5 ± 0·6 × 4·7 ± 0·8	4·0 ± 0·3 × 3·2 ± 0·5	39·0 ± 5·0 × 34·5 ± 3·8	1·4 ± 0·5	7·5 ± 0·6 × 1·9 ± 0·3	2·4 ± 0·5 × 1·6 ± 0·3	Baneth *et al.* (2013)
*H. felis*	*Prionailurus bengalensis*	Korea	N/A	N/A	31 ± 4 × 19 ± 3	N/A	N/A	N/A	Kubo *et al.* (2010)
*Hepatozoon* spp.	*P. bengalensis*	Thailand	9·8 ± 0·4 × 5·2 ± 0·4	N/A	N/A	N/A	N/A	N/A	Salakij *et al.* (2010)
*Hepatozoon* spp.	*Prionailurus planiceps*	Thailand	9·9 ± 1·1 × 4·8 ± 0·6	N/A	N/A	N/A	N/A	N/A	Salakij *et al.* (2008)
*Hepatozoon* spp.	*Leopardus pardalis*	Brazil	7·43 × 4·23	N/A	N/A	N/A	N/A	N/A	Metzger *et al.* (2008)
*Hepatozoon* spp.	*Felis iriomotensis, Felis bengalensis euptilura*	Japan	N/A	N/A	22·3 ± 3·1 × 15·3 ± 2·2	N/A	6·1 ± 0·6 × 2·3 ± 0·2	N/A	Kubo *et al.* (2006)
*Hepatozoon* spp.	*Panthera leo*	Tanzania	N/A	N/A	30 × 23	N/A	N/A	N/A	Averbeck *et al.* (1990)
*Hepatozoon* spp.	*Felis catus*	Israel	N/A	N/A	22 ± 4·8	N/A	N/A	N/A	Klopfer *et al.* (1973)

N/A, data not available.

a refers to gamont-like stage.

Furthermore, multifocal infiltrates of mild to moderate numbers of lymphocytes, macrophages, and rare neutrophils and eosinophils were seen in the heart of three cats. Inflammatory cells were mostly located in the interstitium of subendocardial areas. Only a few intact meronts were surrounded with small numbers of inflammatory cells (Fig. 1B). In the skeletal muscle of two cats, a few areas of interstitium were infiltrated with small numbers of lymphocytes and macrophages. However, no inflammatory response was observed around the meronts. In the severely affected cat (170/16), interalveolar and interlobular lung septa were distended and thickened with moderate numbers of inflammatory cells, mostly macrophages and lymphocytes. Multifocal areas of peribronchial and perivacular oedema were noticeable. Despite the presence of meronts confirmed by histopathology, no other lesions associated with *Hepatozoon* infection were found in the heart, spleen and muscle tissue of the cat. Moreover, four males and 17 semi- and fully engorged females of *Ixodes ricinus* were found on this cat, and no parasitic stages were observed in haemolymph smears of any of those ticks. This might be because the ticks did not feed on an infected animal in the nymphal stage or they were not examined after enough time to allow the development of the protozoan oocysts.

### Identification and molecular characterization of Hepatozoon spp.

A total six (67%) out of the nine wild cats collected were positive for *Hepatozoon* spp. by PCR using the genus-specific primers (H14Hepa18SFw and H14Hepa18SRv). The heart of five wild cats, lungs of four, spleen of three and brachial muscle of two cats scored positive (Table 2). For phylogenetic analyses, samples of all the six positive cats were additionally tested with another PCR protocol that amplifies a larger segment (approximately 1700 bp) of the 18S rRNA gene of *Hepatozoon* spp. This PCR produced positive results in frozen samples of five cats, but not in paraffin-embedded tissues of a cat positive in the first PCR. However, only three high-quality sequences were obtained and used for phylogenetic analyses. None of the samples tested were PCR positive for *T. gondii* or *Sarcocystis* spp.

Analysis and BLAST search of the short sequence (572 bp) obtained from one wild cat yielded 100% identity to the sequences of *H. felis* previously described in Iriomote cat (*Prionailurus iriomotensis*), Tsushima leopard cat (*Prionailurus bengalensis euptilurus*) and their ticks from Japan (GenBank^®^ accession numbers: AB771513, AB771568 and AB983435, respectively). Sequences of the remaining five PCR positive cats (five of the shorter and three of the longer fragment) were identical to each other, but distinct from the sequences of *H. felis*. The short sequences displayed a 96% identity to the most closely related sequences of *H. felis* haplotypes (JN584475, HQ829439, HQ829444, AY628681, AY620232, JN123435) detected in domestic and various species of wild cats. Regarding the longer sequences herein generated (1669 bp), they shared a 97% identity with the *H. felis* (AY628681, AY620232) sequences from domestic cats (*Felis catus*), and 96% identity to *H. canis* (AY150067, KC138531) and *Hepatozoon ayorgbor* (EF157822) sequences previously found in the red fox (*Vulpes vulpes*), domestic cat (*Felis catus*) and the ball python (*Python regius*), respectively.

***Description***

*Hepatozoon silvestris* sp. nov.

(Figures 1–3).

**Meront**: Round to oval with a mean length of 31·7 ± 4·2 *µ*m (22·1–38·7 *µ*m) by 22·0 ± 4·6 *µ*m (11·9–28·3 *µ*m) and a shape index (length/width ratio) of 1·4 (*n* = 16). Meronts were enveloped by a relatively thick capsule (mean width 1·1 ± 0·3 *µ*m, 0·51–1·79 *µ*m, *n* = 7), which separated them from the surrounding tissues. Early meronts were smaller and comprised light basophilic to amphophilic amorphous material that sometimes contained small vacuoles and peripherally arranged nuclei. Two types of developing meronts were observed. Typical wheel spoke-shaped meronts contained approximately 20–30 small, round to oval micromerozoites arranged in a circle around the basal material mass; and a second type of meronts contained 2–8 larger and elongate macromerozoites dispersed within the meront, and sometimes circularly arranged leaning with the longer side against the meront wall. Mature meronts were characterized by numerous merozoites irregularly scattered across the light basophilic amorphous material. Micromerozoites were 4·2 ± 1·1 *µ*m × 2·5 ± 0·4 *µ*m (2·6–6·3 *µ*m × 1·9–3·3 *µ*m, *n* = 23) with a shape index of 1·7, and macromerozoites measured 6·0 ± 1·0 *µ*m × 3·2 ± 0·7 *µ*m (4·3–7·8 *µ*m× 2·5–4·7 *µ*m, *n* = 9) with a shape index of 1·9. Round to oval dark condensed micromerozoite nuclei were 2·6 ± 0·3 *µ*m (2·0–3·5 *µ*m, *n* = 58), whereas macromerozoite nuclei were ellipsoid to rectangular and measured 4·0 ± 0·3 *µ*m in length (3·5–4·4 *µ*m, *n* = 8).

**Gamont-like stage**: Elongated, slightly bended at the ends, and surrounded with a delicate capsule. The mean size was 11·7 ± 0·5 *µ*m × 5·2 ± 0·7 *µ*m (11·0–12·7 *µ*m × 4·1–6·6 *µ*m, *n* = 11). The cytoplasm was light basophilic, sometimes with darker granules posterior and anterior to the centrally situated, purple to dark kidney-shaped and compact nucleus. The nucleus measured 6·3 ± 1·3 *µ*m × 3·0 ± 0·8 *µ*m (4·1–7·8 *µ*m × 2·0–4·7 *µ*m, *n* = 11). Shape index of gamont-like stage and its nucleus was 2·3 and 2·1, respectively.

***Taxonomic summary***

***Type-host*:** *Felis silvestris silvestris* Schreber, 1777

***Other hosts*:** unknown

***Vector***: unknown

***Site of infection*:** Meronts and gamont-like stages were observed in heart, spleen and skeletal muscle tissue by cytology and histopathology. Likewise, lungs and spleen were positive by PCR and sequencing

***Type-locality*:** Bihać (44·8119628N, 15·8685645E) – Bosnia and Herzegovina

***Other localities*:** Bosanski Petrovac (44·550306N, 16·364375E), Gornji Vakuf (43·9375436N, 17·5880462E) – Bosnia and Herzegovina

***Prevalence*:** Five out of nine (56%) *F. silvestris silvestris* were positive

***Type-material*:** Spleen and heart smears and histology slides from the type-host are deposited in the parasites collection of the Pathology Museum, Faculty of Veterinary Medicine, University of Sarajevo, Bosnia and Herzegovina (accession numbers: VFS-PAT-OP152/16-1, VFS-PAT-OP152/16-2, VFS-PAT-OP152/16-3)

***Other material*:** DNA samples are kept at the Institute of Parasitology, University of Veterinary Medicine Vienna, Austria

***Representative DNA sequences*:** Two sequences representing a 572 and 1669 bp fragment of the 18S rRNA gene have been deposited in the GenBank^®^ database and are available under accession numbers KX757031 and KX757032, respectively

***ZooBank registration*:** In accordance with section 8.5 of the *International Code of Zoological Nomenclature* (ICZN), details of the new species have been submitted to ZooBank with the Life Science Identifier (LSID) urn:lsid:zoobank.org:pub:FB2935A0-617E-4E55-8C32-543493A5FD61

***Etymology*:** The species name follows the type-host species name, it is used as Latin adjective (*silvestris* = from forest, sylvan)

***Remarks*:** Based on histopathological findings, spleen and heart cytology, morphological and morphometric characteristics of developmental stages and finally molecular and phylogenetic analyses, the parasite reported in this study represents a new species, herein described as *H. silvestris* sp. nov. The life stages observed in the stained spleen and heart touch imprints of two cats (Figs 1A and 3A) resembled mature *Hepatozoon* gamonts based on their structure, shape, size and presence of the capsule. However, these forms were found only extracellularly, and not in blood cells, which are specific for gamonts. Consequently, it is difficult to explicitly state whether they represent gamonts released after blood cell membrane damage or even free zoites released from tissue cysts, which could be similar in appearance (Baneth and Shkap, 2003; Baneth *et al.* 2007). Therefore, we refer to these stages of both *Hepatozoon* spp. identified in this study as ‘gamont-like stages’.

Of all *Hepatozoon* species described in carnivores, *H. silvestris* sp. nov. is morphologically and genetically most similar to *H. felis*, but the following features can be used to distinguish the two species: (1) Meronts of *H. silvestris* sp. nov. have thinner capsule; (2) Wheel spoke-shaped meronts of *H. silvestris* sp. nov. contain 20–30 micromerozoites, whereas *H. felis* meronts contain 10–15 micromerozoites; (3) Micromerozoites of *H. silvestris* sp. nov. are smaller and round to oval in shape; in *H. felis*, micromerozoites are larger, rectangular or triangular in shape and oriented perpendicularly to the meront wall.

### Phylogenetic analyses

**Fig. 4.**
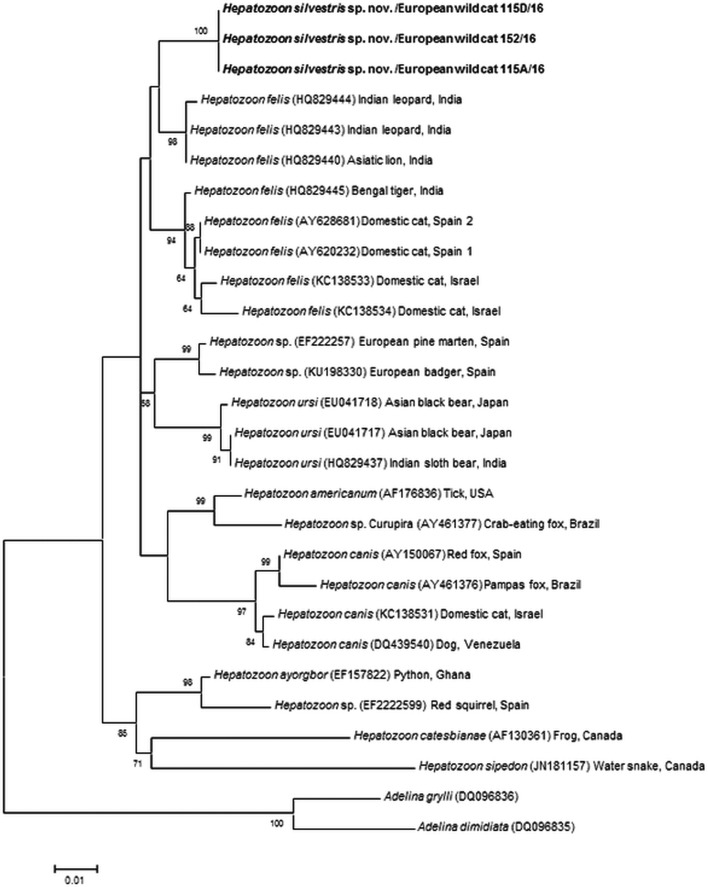
Maximum Likelihood (ML) bootstrap tree with 18S rRNA nucleotide sequences of *Hepatozoon silvestris* sp. nov. (1669 bp) from this study compared to other *Hepatozoon* sequences deposited in GenBank^®^ database. The tree with the highest log likelihood (−3128·227) is shown. Accession numbers (in brackets), host and origin for each sequence analysed are indicated. Bootstrap values based on 1000 replicates are indicated at the nodes (only values over 50% are included). The sequences generated in this study are presented in bold.

**Fig. 5.**
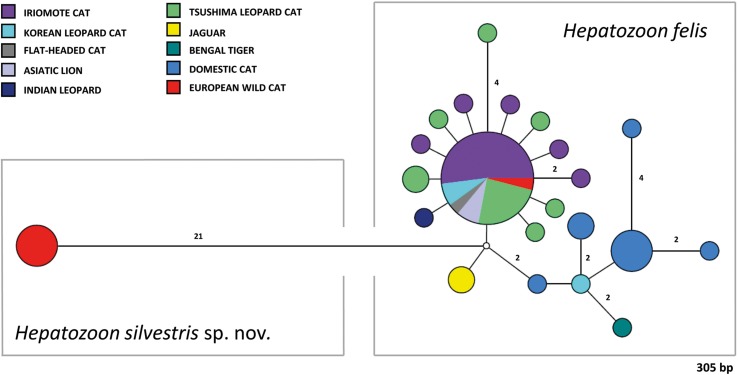
Median-Joining network of the 18S rRNA gene of *Hepatozoon felis* and newly described *Hepatozoon silvestris* sp. nov. (572 bp) constructed using the Network software. The size of the circles in the network is proportional to the number of sequences sharing the same haplotype. Numbers along branches indicate the number of substitutions between haplotypes.

The overall topology of NJ, ML and MP trees inferred based on the total alignment of 28 sequences of *Hepatozoon* spp. (945 bp), including sequences of *Adelina grylli* (DQ096836) and *Adelina dimidiata* (DQ096835) as outgroups, showed the similar branching pattern and indicated the existence of three clades formed by *Hepatozoon* spp. from European and Asian felid hosts (Fig. 4). The sequences of *H. silvestris* sp. nov. found in European wild cats fall within the first clade (100% bootstrap by all methods used), whereas the remaining two clades contain sequences of *H. felis* (or closely related species) from Indian wild cats and domestic cats from Israel and Spain. The Median-Joining network generated using the partial 18S rRNA sequences obtained in this study along with 51 sequences of *H. felis* retrieved from the GenBank^®^ database displayed similar pattern with two well-separated clades (Fig. 5). The first clade contains sequences of *H. felis* representing 21 different haplotypes identified in domestic and wild cats (including a wild cat from this study), whereas the second clade exclusively contains sequences of *H. silvestris* sp. nov. from European wild cats (Fig. 5).

## DISCUSSION

Our study reports the first detection of *Hepatozoon* spp. in European wild cats and clearly indicates the existence of two morphologically and genetically distinct species, namely *H. felis* and the newly characterized *H. silvestris* sp. nov. The phylogenetic tree herein generated shows that *H. silvestris* sp. nov. clusters with other *Hepatozoon* spp. from carnivores such as *H. canis, H. americanum* and *H. felis*, and would also fall within Clade A (Kvičerová *et al.* 2014) or the narrower *Hepatozoon* genus proposed by another study addressing the different lineages in the genus (Karadjian *et al.* 2015). Moreover, sequences of *H. felis* from domestic and wild cats used for phylogenetic analysis in this study, formed two highly supported clades indicating that probably only one of them could be referred to as *H. felis*, and another one may represent a different species as already suggested (Pawar *et al.* 2012).

Microscopically, two different life stages were observed from the infected animals by cytology and histopathology. Meronts were the most frequently encountered protozoan stage and different developing types were found to be present in heart, lungs, spleen and skeletal muscle tissue. Regarding the morphometric characteristics, meronts of *H. silvestris* sp. nov. observed in tissue sections differ in size, shape and merozoites arrangement compared with those of *H. felis* or closely related species reported from domestic and wild cats (Table 2). In general, meronts of *H. silvestris* sp. nov. (31·7 ± 4·2 *µ*m × 22·0 ± 4·6 *µ*m) were smaller in size than the *H. felis* meronts (36·4 ± 5·1 *µ*m × 31·3 ± 5·1 *µ*m) found in this study, and also to those described in domestic cats, which were 39·0 ± 5·0 *µ*m × 34·5 ± 3·8 *µ*m (Baneth *et al.* 2013). However, the mean size of the *H. silvestris* sp. nov. meronts was larger in comparison to those of *Hepatozoon* spp. reported from the Iriomote cat, leopard cat (*P. bengalensis*) and domestic cat which were 22·3 ± 3·1 *µ*m × 15·3 ± 2·2 *µ*m (Kubo *et al.* 2006) and 22 ± 4·8 *µ*m in length (Klopfer *et al.* 1973), respectively, and similar in size to the meronts observed in the myocardium of the leopard cat (31 ± 4 *µ*m × 19 ± 3 *µ*m, Kubo *et al.* 2010) and lion (*Panthera leo*) (30  ×  23 *µ*m in length, Averbeck *et al.* 1990). The differences in the sizes of meronts between the studies may be due to different fixation procedures used for specimens preparation, errors in measurement or even affiliation to different species or strains of *Hepatozoon* as already suggested (Baneth and Shkap, 2003; Kubo *et al.* 2010). Histopathology revealed the presence of two types of developing meronts in wild cats infected with *H. silvestris* sp. nov. similar to those described in *H. canis* (Baneth *et al.* 2007). Meronts with a typical wheel spoke pattern containing 20–30 small round to oval micromerozoites, and a second type containing 2–8 larger macromerozoites either dispersed within the meront or evenly lined up with longer side along the meront wall. However, only one meront type of *H. felis* with 10–15 circularly arranged large, rectangular or triangular micromerozoites oriented perpendicularly to the meront wall was observed in tissue sections of the heart, lungs and spleen (Fig. 3C). This type of meront has not been found in *H. felis*-infected domestic cats (Baneth *et al.* 2013), but similar structures have been described in the heart of wild cats in Japan (Kubo *et al.* 2006). Furthermore, the capsule that surrounds the meronts of *H. silvestris* sp. nov. was thinner (1·1 ± 0·3 *µ*m) than the capsule of *H. felis* (1·4 ± 0·3 *µ*m).

Meronts of *H. silvestris* sp. nov. were found in cardiac and skeletal muscle tissue, but the parasite's DNA was also detected by PCR in the lungs and spleen of infected animals. Predilection to muscle tissue has also been reported in domestic and wild cats infected with *H. felis* (Klopfer *et al.* 1973; Averbeck *et al.* 1990; Beaufils *et al*. 1998; Kubo *et al.* 2006, 2010; Baneth *et al.* 2013) and dogs and wildlife infected with *Hepatozoon americanum* (Vincent-Johnson *et al.* 1997). This is substantially different from *H. canis* where infection is associated with the occurrence of meronts in haemolymphoid tissues (e.g. spleen, lymph nodes and bone marrow) and not in muscle tissue (Vincent-Johnson *et al.* 1997; Baneth *et al.* 2007, 2013). However, *H. americanum* produces ‘onion skin’ cysts in skeletal muscles, which are much larger and different in appearance than *H. silvestris* sp. nov. or *H. felis* meronts, and give rise to severe pyogranulomatous myositis, muscular hyperaesthesia and periostal reaction (Vincent-Johnson *et al.* 1997; Ewing and Panciera, 2003). Nevertheless, mild myocarditis with multifocal infiltrates of lymphocytes, macrophages, and rare neutrophils and eosinophils were observed in the heart of three cats infected with *H. silvestris* sp. nov., but not in the cat severely infected with meronts of *H. felis*. Similar lesions have been described in African and Asian wild cats, spotted hyenas (*Crocuta crocuta*) and Pampas grey fox (*Lycalopex gymnocercus*) infected with *H. felis* or closely related species (Kubo *et al.* 2006, 2010; East *et al.* 2008; Giannitti *et al.* 2012). Moreover, elevated activities of serum lactate dehydrogenase and creatine kinase have been reported in domestic cats with hepatozoonosis indicating muscular tissue damage (Baneth *et al.* 1998; Perez *et al.* 2004).

The gamont-like stage of *H. silvestris* sp. nov. is bigger in size (11·7 ± 0·5 *µ*m × 5·2 ± 0·7 *µ*m) than that of *H. felis* (10·5 ± 0·4 *µ*m × 4·4 ± 0·4 *µ*m) and it has a more compact nucleus that is longer and narrower (6·3 ± 1·3 *µ*m × 3·0 ± 0·8 *µ*m) than the less densely appearing nucleus of *H. felis* (4·7 ± 0·3 *µ*m × 4·4 ± 0·3 *µ*m). However, the gamont-like stage of *H. felis* is very similar in appearance and size to the gamonts of *H. felis* described in stained blood smears from domestic cats (10·5 ± 0·6 *µ*m × 4·7 ± 0·8 *µ*m, Baneth *et al.* 2013), and bigger than those of *Hepatozoon* spp. from the ocelot (*Leopardus pardalis*) (7·43 × 4·23 *µ*m), flat-headed cat (*Prionailurus planiceps*) (9·9 ± 1·1 *µ*m × 4·8 ± 0·6 *µ*m^2^) and leopard cat (9·8 ± 0·4 *µ*m × 5·2 ± 0·4 *µ*m) (Metzger *et al.* 2008; Salakij *et al.* 2008, 2010).

The vectors of feline hepatozoonosis are unknown, and in general data regarding the blood sucking invertebrates parasitizing wild cats in Europe are scarce. A recent study on wild cats in Romania showed that *I. ricinus* was the dominant tick species infesting wild cats (Gallusová *et al.* 2016), which is in agreement with our study in which it was also the only species found. However, no evidence of infection with *Hepatozoon* oocysts was found in ticks collected from the European wild cat. Furthermore, other alternative routes of the parasite's transmission should be considered (Baneth *et al.* 2013). The detection of *H. silvestris* sp. nov. meronts in the cardiac and skeletal muscle tissues of the infected animals and, molecularly, in lungs and spleen, along with the marked predatory behaviour of wild felids, might suggest that the infection is transmitted by carnivorism. This could be also supported by the lack of detection of gamonts in circulating leucocytes, such as neutrophils and monocytes.

### Concluding remarks

In this study, we described a new species of *Hepatozoon*, clearly distinct from *H. felis*, found to infect European wild cats in Bosnia and Herzegovina. In addition, the study reports the occurrence of *H. felis* for the first time in wild cats in Europe, extending the host and distribution range of this feline parasite. The European wild cats and domestic cats are two close relatives, which may live in sympatry, interbreed and even produce fertile offspring (Mattucci *et al.* 2013). As a consequence of the close association between wild and domestic cats, both species may share pathogens and be at risk of the same infections (Veronesi *et al.* 2016*b*). Thus, future studies should also include domestic cats from the areas where the wild cats positive for *H. silvestris* sp. nov. were found, in order to investigate their potential ability to serve as intermediate hosts of this newly described species. Furthermore, identification of its definitive host(s) and experimental transmission studies are required for elucidating the life cycle and possible alternative routes of transmission.
